# A comprehensive analysis of vitamin A deficiency in China from 1990 to 2021, examining temporal trends and demographic influences

**DOI:** 10.3389/fnut.2025.1681583

**Published:** 2025-10-09

**Authors:** Yue Du, Feng Jiang, Tianyao Lei, Chenxia Juan, Yan Mao

**Affiliations:** ^1^Jiangsu Province Hospital of Chinese Medicine, Affiliated Hospital of Nanjing University of Chinese Medicine, Nanjing, China; ^2^Obstetrics and Gynecology Hospital of Fudan University, Shanghai, China

**Keywords:** vitamin A deficiency, global burden of disease study, age-period-cohort analysis, temporal trends, frontier analysis

## Abstract

**Background:**

Vitamin A deficiency (VAD) remains a critical public health burden in China, particularly affecting children and reproductive-age women. A comprehensive analysis of its long-term trends is essential for guiding nutritional interventions.

**Methods:**

Utilizing data from the Global Burden of Disease (GBD) 2021 study covering 1990 to 2021, we examined age-standardized incidence, prevalence, disability-adjusted life years (DALYs), and sociodemographic factors influencing VAD in China. Joinpoint regression detected temporal inflection points, and Bayesian age-period-cohort (BAPC) modeling forecasted incidence up to 2040. Frontier analysis correlated burden with the socio-demographic Index (SDI) across 204 countries.

**Results:**

In 2021, China reported 23.19 million new VAD cases, with higher age-standardized incidence in females (2,094.3/100,000) versus males (1,821.07/100,000). A bimodal age distribution peaked in children (<14 years, male-predominant DALYs) and young adults (20–34 years, female-predominant incidence). From 1990–2021, age-standardized rates declined by 81.2% (incidence) and 81.2% (prevalence), surpassing global reductions. Critically, a 2013 inflection point reversed sex disparities: male burden exceeded females before 2013, but female rates dominated thereafter. Sub-Saharan Africa experienced the highest burden globally, with a negative correlation between SDI and VAD incidence (R^2^ = 0.72, *p* < 0.001). BAPC projections indicate persistent declines through 2040, but males will retain higher incidence than females.

**Conclusion:**

Despite substantial progress, VAD persists with evolving sex-age disparities. The 2013 sex burden reversal and dual vulnerability of children/young adults necessitate sex-stratified interventions. Integration of supplementation programs with socioeconomic development is vital for eliminating VAD in China and high-burden regions.

## Introduction

Vitamin A deficiency (VAD) poses a critical global public health burden, particularly in low-resource settings, due to its profound impact on vision, immunity, and cellular function (1). As an essential micronutrient, vitamin A is vital for maintaining epithelial integrity, supporting adaptive immunity, and preventing xerophthalmia and infectious morbidity (2, 3). Globally, VAD contributes significantly to child mortality, susceptibility to infections like measles and diarrhea, and irreversible blindness, disproportionately affecting young children and pregnant women in developing regions (4, 5). In China, rapid socioeconomic development and nutrition transitions have reshaped dietary patterns, yet disparities persist (6). Rural populations, economically disadvantaged groups, and children under five remain at elevated risk due to limited access to vitamin A-rich foods and suboptimal supplementation coverage (7).

The Global Burden of Disease (GBD) study assesses the health impact of VAD by examining its prevalence, mortality rates, and disability-adjusted life years (DALYs), highlighting ongoing disparities (8). GBD 2021 data enables granular analysis of VAD trends across demographics, geographies, and time-crucial for identifying high-risk subgroups and guiding interventions (9). While prior studies highlight declining global VAD prevalence, systematic assessments of its long-term burden in China-integrating incidence, DALYs, and temporal drivers-remain limited. Economic reforms and initiatives (e.g., vitamin A supplementation programs) have reduced severe deficiency (10), but subclinical VAD and regional disparities endure.

This study explored key gaps by leveraging GBD 2021 data to analyze three decades (1990–2021) of VAD burden across China. Further investigation identified significant trend shifts and worldwide distribution in incidence, prevalence, and DALYs of VAD. Then, frontier and correlation analyses robustly linked sociodemographic index (SDI) to VAD burden. Finally, Bayesian age-period-cohort (BAPC) projection estimated incidence through 2040. By stratifying outcomes by age, sex, and region, this work aims to inform targeted nutrition policies, optimize supplementation strategies, and accelerate progress toward VAD elimination in high-risk populations.

## Materials and methods

### Data sources

This research employed data from the GBD 2021, an extensive and publicly accessible resource created by the Institute for Health Metrics and Evaluation (IHME) (11). The GBD framework offers standardized estimates of incidence, prevalence, DALYs and years lived with disability (YLDs) for numerous diseases and injuries across 204 countries and territories, including China, from 1990 to 2021 (12, 13). In this study, we obtained VAD-specific data from the GBD Results Tool, encompassing all-age counts and age-standardized rates per 100,000 population for incidence (ASIR), prevalence (ASPR), and DALYs. GBD estimates are generated through a robust statistical modeling process that integrates data from various sources such as household surveys, censuses, disease registries, and vital statistics, ensuring adjustments for data quality and comparability across different times and regions. The analysis utilized China-specific data from primary sources such as the China Disease Surveillance Points System, national nutrition and health surveys, population censuses, and additional peer-reviewed epidemiological studies (14). Age-standardization utilized the GBD world population standard to enable cross-population comparisons. All estimates incorporated 95% uncertainty intervals (UIs) derived from 1,000 Monte Carlo simulation draws from posterior distributions, capturing uncertainty from sampling and modeling processes.

### Descriptive analysis

We first performed a descriptive analysis to quantify the burden of VAD in China from 1990 to 2021 using GBD 2021 estimates. Key indicators comprised the incidence, prevalence, and DALYs, expressed as both number and age-standardized rates per 100,000 population. Estimates were categorized by sex, age group, and year to examine specific population distributions and temporal trends. Age-standardization utilized the GBD global standard population to enable temporal and gender comparisons. In 2021, visualizations of age- and sex-specific VAD patterns identified childhood and young adulthood as the population segments with the highest burden. The study evaluated temporal trends in all-age numbers and age-standardized rates over a 32-year period, focusing on key changes in incidence, prevalence, and DALYs. National trends in China were contextualized using extracted GBD metrics for comparison. Descriptive statistics included 95% UIs to represent variability from data sources and model simulations, following the GBD methodology (15, 16). The incidence and prevalence rates of VAD were visualized using heatmaps and world maps, created with the “ComplexHeatmap” (17) and “maps” (18) packages in R (version 4.5.1).

### Sociodemographic index (SDI)

SDI is a composite indicator of income per capita, average educational attainment, and total fertility rates (19). The values of SDI range from 0 (worst) to 1 (best), reflecting the degree of socio-development status (20). We assessed the association between SDI and the incidence rate in VAD.

### Frontier analysis

Frontier analysis was utilized to benchmark VAD burden by comparing countries and territories with the top performers (21). This approach highlights pioneering countries and territories, establishing benchmarks and objectives for others to follow. We calculated the “effective difference” (22) for each country and territory, representing the SDI-adjusted gap between the actual and potential VAD burden.

### Bayesian age-period-cohort (BAPC) analysis

We utilized a BAPC model to project sex-specific trends in age-standardized incidence rates of VAD in China through 2040. The BAPC model utilized the BAPC and integrated nested Laplace approximations (INLA) packages in R, employing INLA for efficient Bayesian inference (23). This method enables robust estimation by considering temporal dependencies and smoothing effects across age, period, and cohort dimensions, making it ideal for public health forecasting with GBD-type longitudinal data. The BAPC model was applied to GBD 2021 estimates from 1990 to 2021, categorized by sex and 5-year age intervals. The uncertainty bands, 95% Bayesian credibility intervals (CrIs), around the point forecasts represent the statistical uncertainty inherent in this extrapolation, capturing the range within which future values are expected to fall based on the model and historical variability. The CrIs, derived from posterior distributions using 1,000 simulations, are displayed with central estimates in the figures and tables (24).

## Results

### Burden of VAD in China, 2021

In 2021, VAD remained a significant public health concern in China, with notable disparities between sexes. There were 23.19 million new VAD cases reported, with males having a lower age-standardized incidence rate of 1,821.07 (95% UI: 1,411.59-2,309.28) per 100,000 compared to females at 2,094.3 (95% UI: 1,719.55-2,542.25) per 100,000. The prevalence followed a similar pattern, with over 23.16 million people affected and females again showing a higher age-standardized rate. The total burden, quantified in DALYs, was 39,094. Males experienced a greater burden, as indicated by a significantly higher age-standardized DALY rate compared to females. YLDs had a minimal impact on the overall burden, yet the YLDs rate was significantly higher in males (3.78 per 100,000; 95% UI: 2.33–5.6 per 100,000) than in females (3.06 per 100,000, 95% UI: 1.86–4.58 per 100,000). These findings emphasize the ongoing impact of VAD in China and the necessity for gender-specific nutritional and healthcare approaches (Table 1). It was obvious that in 2021, the DALYs attributable to VAD in China were predominantly driven by YLDs, which accounted for nearly 100% of the total burden, reflecting the significant morbidity rather than mortality associated with the deficiency.

**Table 1:** All-age cases and age-standardized incidence, prevalence, and DALYs rates in 2021 for VAD in China.

Measure	All ages cases			Age-standardized rates per 100,000 people		
	Total	Male	Female	Total	Male	Female
Incidence	23,194,527 (20,122,649, 26,881,302)	11,269,440 (8,781,670, 14,451,422)	11,925,086 (9,834,403, 14,210,752)	1951.02 (1673.03, 2272.01)	1821.07 (1411.59, 2309.28)	2094.3 (1719.55, 2542.25)
Prevalence	23,158,474 (20,089,355, 26,846,699)	11,245,036 (8,759,928, 14,426,378)	11,913,438 (9,824,254, 14,197,345)	1947.5 (1669.69, 2268.7)	1816.57 (1406.82, 2304.48)	2091.89 (1716.78, 2540.17)
DALYs	39,094 (24,873, 57,158)	22,223 (14,006, 33,194)	16,871 (10,725, 24,596)	3.44 (2.14, 5.11)	3.78 (2.33, 5.6)	3.06 (1.86, 4.58)
YLDs	39,094 (24,873, 57,158)	22,223 (14,006, 33,194)	16,871 (10,725, 24,596)	3.44 (2.14, 5.11)	3.78 (2.33, 5.6)	3.06 (1.86, 4.58)

### Age and sex patterns of VAD in China, 2021

**Figure 1:**
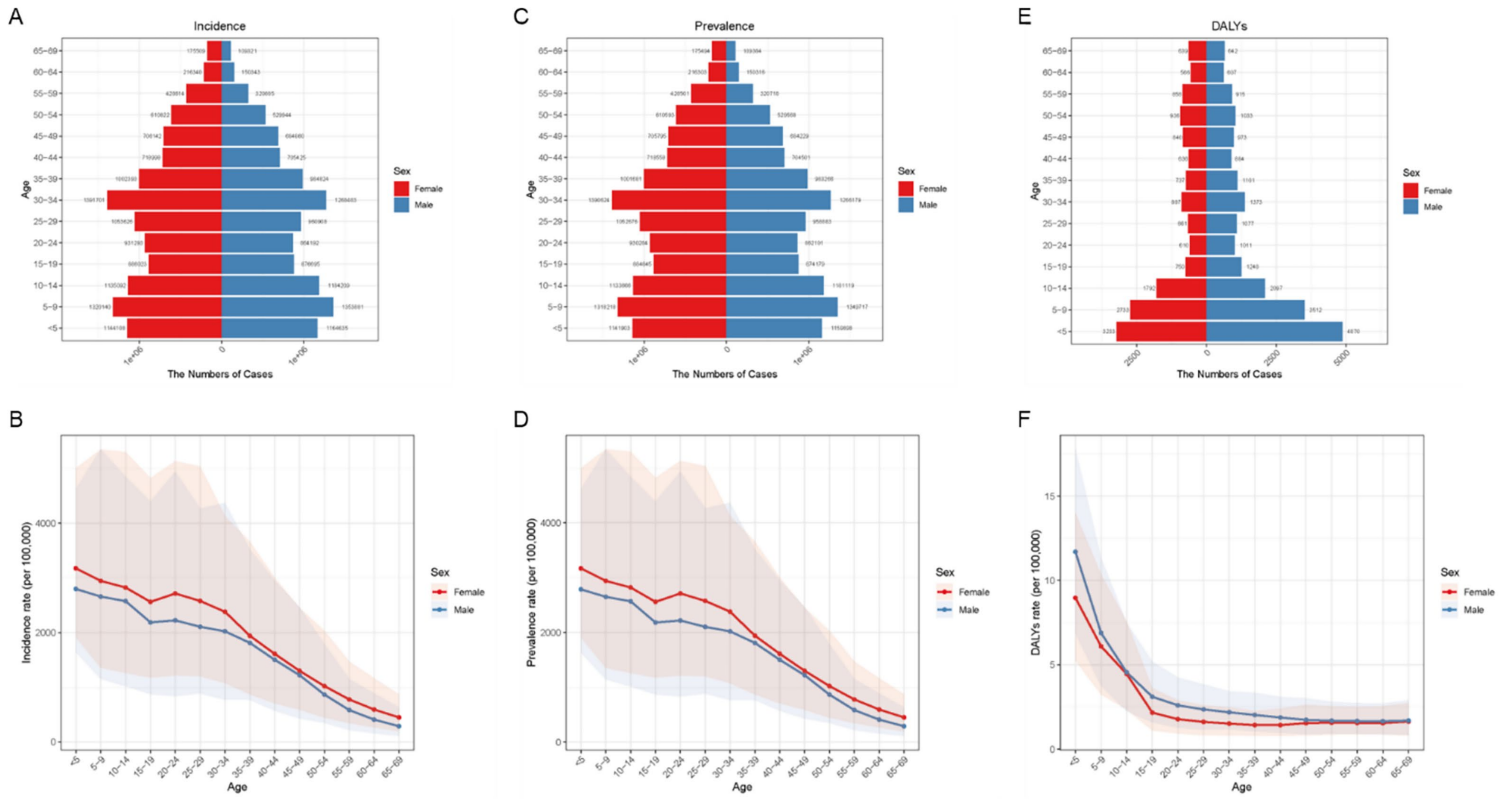
Age and sex distribution of incidence, prevalence, and DALYs rates of VAD in China in 2021. **(A)** Age-specific and sex-specific number of incident cases of VAD. **(B)** Age-specific and sex-specific rate of incidence of VAD per 100,000 people. **(C)** Age-specific and sex-specific number of prevalent cases of VAD. **(D)** Age-specific and sex-specific rate of incidence of VAD per 100,000 people. **(E)** Age-specific and sex-specific number of DALY cases of VAD. **(F)** Age-specific and sex-specific rate of DALYs due to VAD per 100,000 people.

In 2021, China experienced notable age- and sex-related disparities in VAD burden, with bimodal peaks evident among children and young adults. Young adults, especially females aged 30–34, and children, particularly males under 14, exhibited the highest incidence of VAD, representing the majority of new cases (Figure 1A). As shown in Figure 1B, the incidence rate of VAD generally decreases with age. It is worth noting that young people aged 20–24 years show a high age specific incidence rate, reflecting the vulnerability of young people, especially girls. The prevalence is similarly distributed, with a higher burden observed in individuals aged 30–34 and children aged 5–9 (Figure 1C). The disease consistently shows a higher incidence in women across all age groups (Figure 1D). The burden of DALYs mirrored these trends, reaching its peak in children under 14 years old, and the younger the age, the higher the value, with a disproportionate proportion of males (Figures 1E,F).

### Temporal trends and global comparison of VAD burden in China, 1990–2021

**Figure 2:**
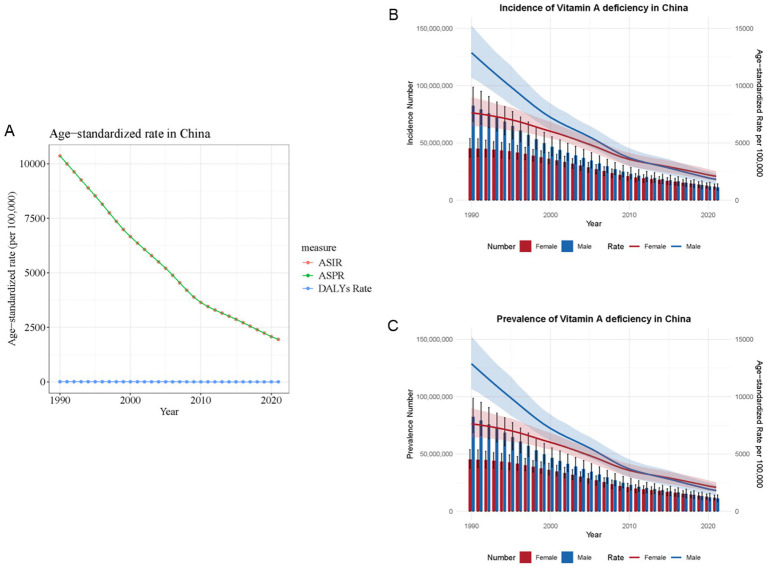
Temporal trends and global comparison of vitamin A deficiency burden in China, 1990–2021. **(A)** China trend in the age-standardized rates of VAD. **(B)** Trends in the number and age-standardized rate of incidence of VAD. **(C)** Trends in the number and age-standardized rate of prevalence of VAD. ASIR, age-standardized incidence rate; ASPR, age-standardized prevalence rate.

From 1990 to 2021, China saw significant reductions in the age-standardized incidence and prevalence rates of VAD. As shown in Figure 2A, China’s age-standardized incidence rate fell markedly from 10,360 to 1,951 per 100,000 population, while the prevalence rate dropped from 10,358 to 1,947. From 1990 to 2021, China’s VAD burden exhibited significant temporal variations, with distinct differences between sexes. The incidence of VAD has declined since 1990 and stabilized around 2013. This trend was more pronounced in males, showing a steeper decline, while females experienced more gradual changes (Figure 2B). A comparable trend was noted in prevalence (Figure 2C). Before 2013, males exhibited higher VAD-related incidence and prevalence rates, but post-2013, these rates became higher in females (Figures 2B,C). These trends indicate changing epidemiological dynamics, particularly around 2013, highlighting the necessity for gender-specific interventions to tackle the shifting burden of VAD.

### Worldwide patterns of VAD are characterized by its incidence, prevalence, and associated DALYs

**Figure 3:**
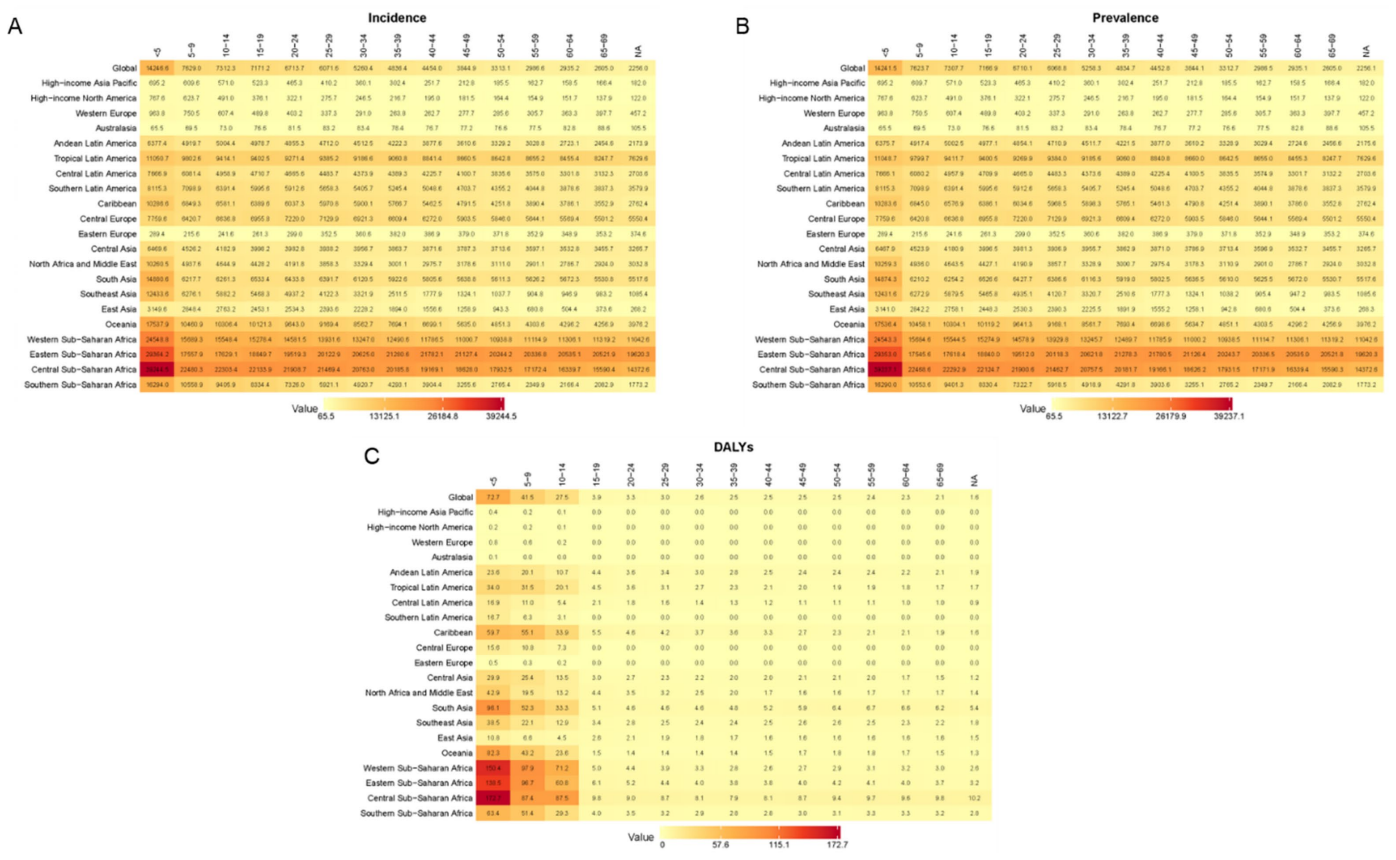
Worldwide patterns of VAD are characterized by its incidence, prevalence, and associated DALYs. Heatmap of the incidence rate **(A)**, prevalence rate **(B)**, and DALYs **(C)** in different countries and age groups.

**Figure 4:**
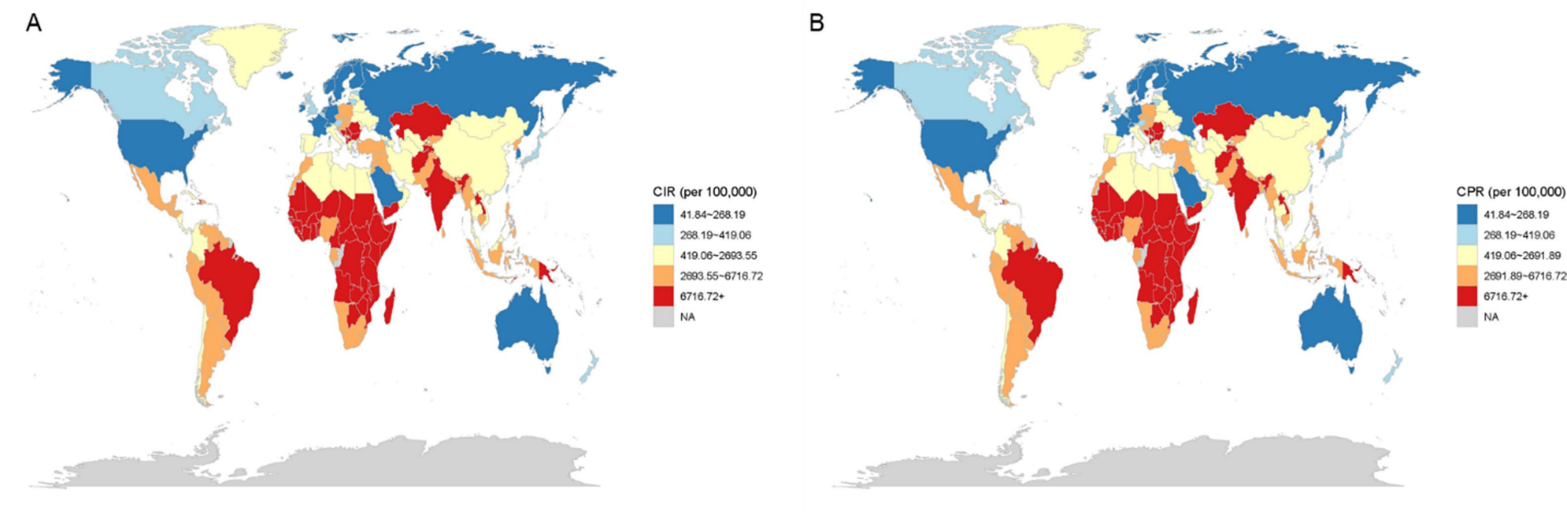
World maps of the incidence rate **(A)** and prevalence rate **(B)**.

From 1990 to 2021, there was a significant decrease in the age-standardized incidence and prevalence rates of the global burden of VAD. The heatmap in Figure 3A showed the incidence rate of each age group in each region. Notably, Central, East, and West Sub-Saharan Africa consistently ranked as the top three regions with the highest incidence rates across all age groups. In these three regions, the incidence rate of patients younger than 5 years old was the highest. Similarly, the age distribution in other regions was mostly consistent with these three regions. Also, the distribution of prevalence of VAD (Figure 3B) was similar to that of incidence. The global map in Figure 4 further reported the incidence rate and prevalence of VAD, which was mutually supportive with the results reported in Figure 3.

### Correlation between incidence of VAD and socio-demographics

**Figure 5:**
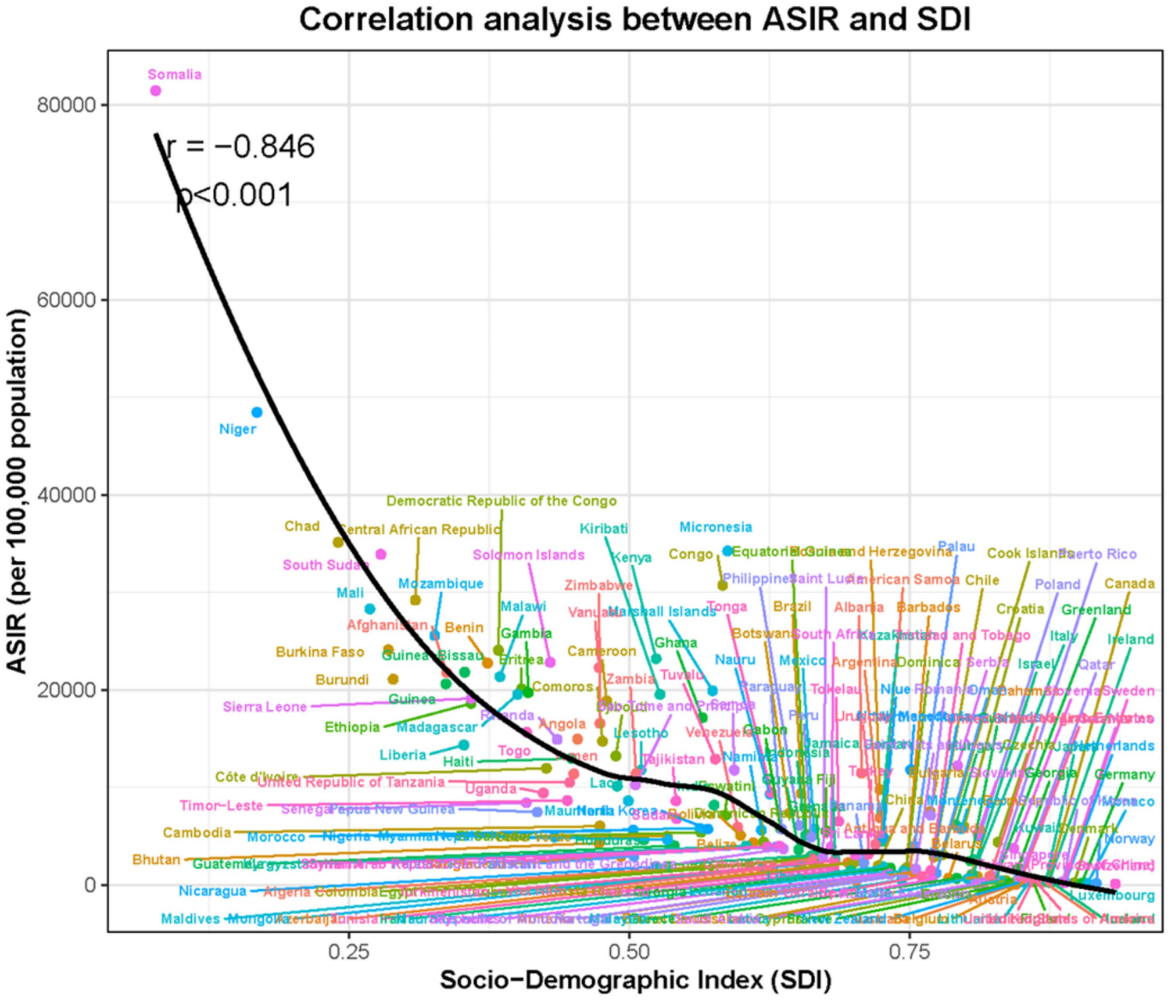
The correlation analyses of incidence rate and SDI for VAD from 1990 to 2021.

We created scatter plots to examine the impact of SDI on VAD incidence trends, depicting changes in SDI and ASIR across 204 countries over the last 32 years. A negative correlation between SDI and the ASIR of VAD was observed across various countries, indicating that higher SDI levels consistently corresponded with lower VAD incidence (Figure 5).

### Frontier analysis on VAD DALYs

**Figure 6:**
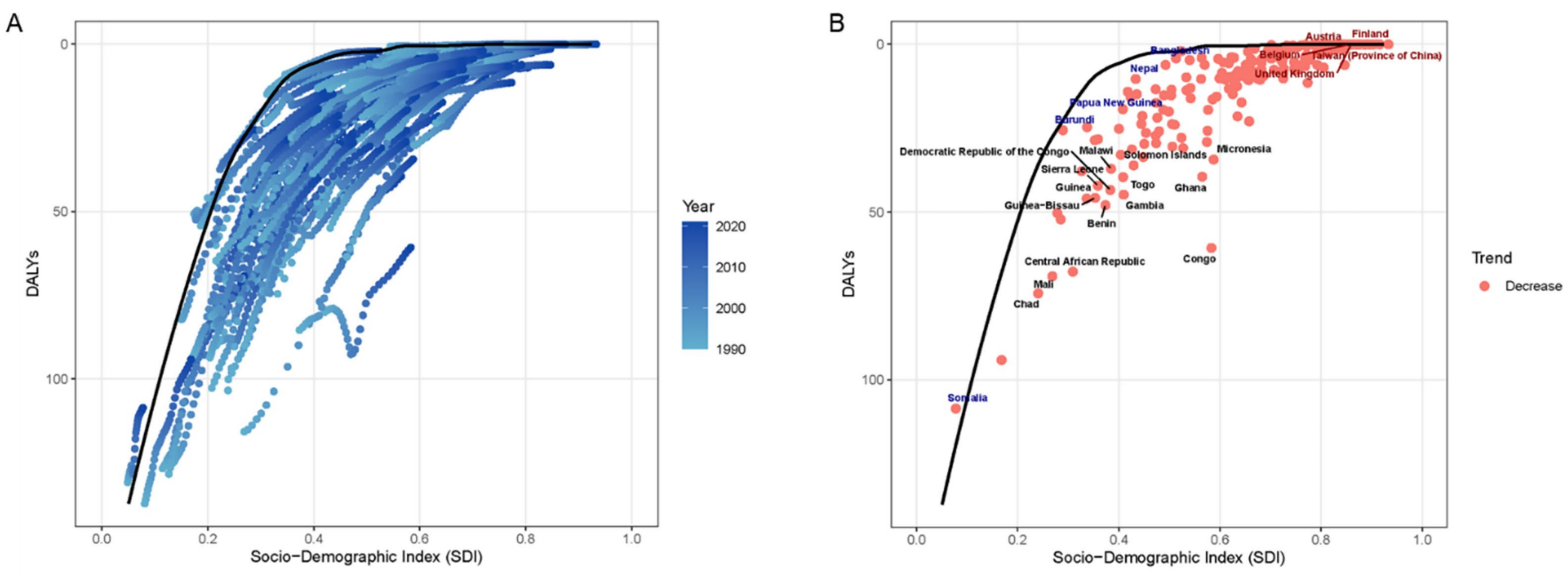
The relationship between SDI and ASR for DALYs in the context of VAD by frontier analysis. **(A)** The progression of years, ranging from 1990 to 2021. Scatterplot shows temporal evolution of each country’s DALYs relative to SDI, with LOESS-derived frontier line representing optimal performance. **(B)** Each dot signifies a specific country or territory for the year 2021. Highlighted are the 15 countries with the greatest disease burden (black text), the five countries with low SDI and disease burden (blue text), and the five countries with high-SDI and disease burden (red text).

A comprehensive frontier analysis of SDI and age-standardized rates (ASRs) of VAD from 1990 to 2021 across 204 countries and territories revealed distinct trends. As the SDI value increased from 0.0 to 1.0, there was a general decrease in ASR for VAD, shown by a shift from lighter to darker shades over the years, indicating a reduction in DALYs (Figure 6).

### Projections of VAD incidence in China through 2040 based on BAPC modeling

**Figure 7:**
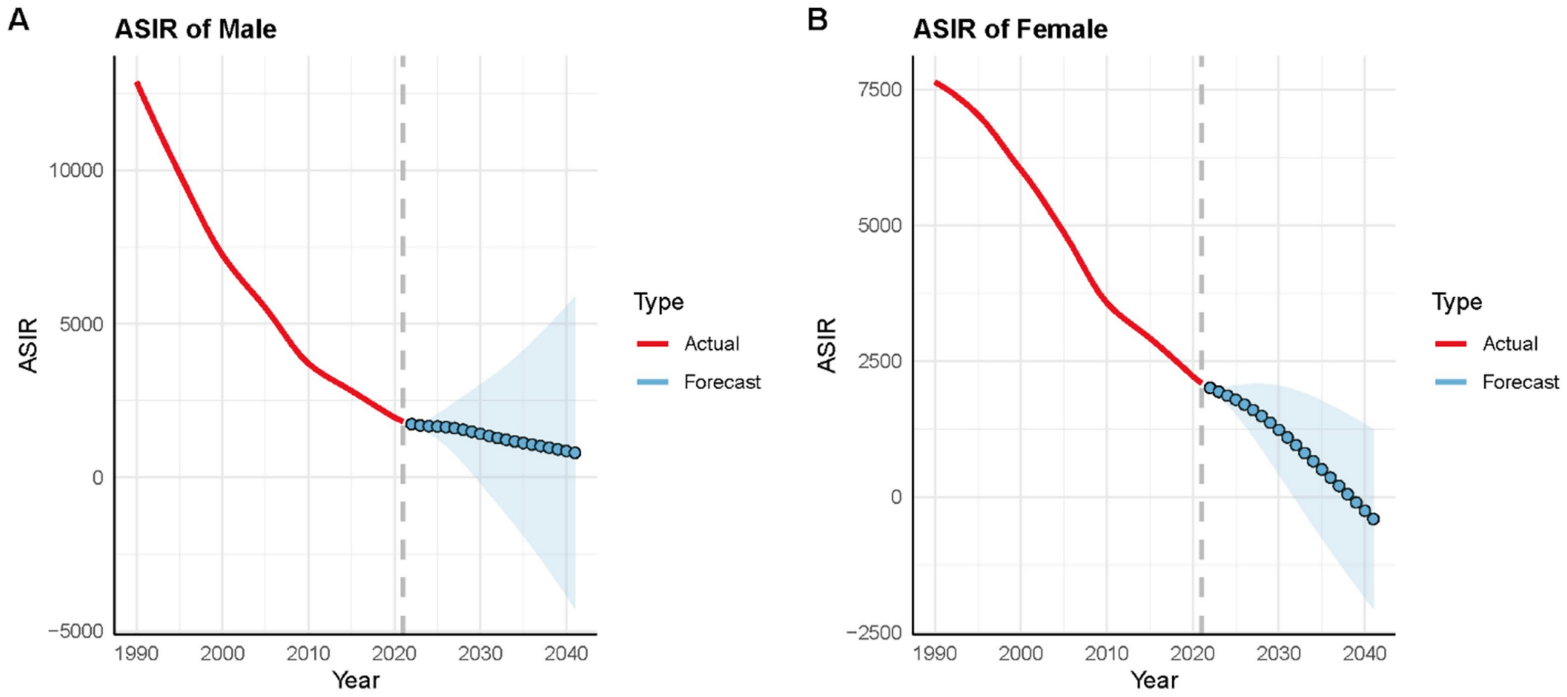
BAPC model projections of VAD incidence rates in China for males and females. Shaded areas around the lines represent % Bayesian credibility intervals. **(A)** Male incidence rates; **(B)** Female incidence rates.

Figure 7 illustrates the BAPC projections for VAD incidence rates in China for both sexes through 2040. The analysis indicated that although declines in this indicator were anticipated for both genders, females were projected to experience these reductions more swiftly. Incidence rates decreased for both sexes, with a more pronounced decline in females, leading to a gradual widening of the sex gap over time (Figures 7A,B). Although overall improvements are expected, males are projected to consistently have a higher incidence rate than females during the projection period.

### Discussion

Utilizing GBD 2021 data, this study provides a comprehensive evaluation of long-term trends and future projections of VAD in China from 1990 to 2021. It is obvious that age-standardized incidence and prevalence rates of VAD in China both reduced significantly in the past decades. It is noteworthy that the declining trend of VAD in China reported by the GBD study is consistent with the directionality reported in nationally representative surveys, such as the China Nutrition and Health Survey (CNHS). For instance, CNHS data has indicated a significant improvement in vitamin status among Chinese children over the past decades, coinciding with rapid economic growth and national nutritional interventions (25, 26). While direct numerical correlations may vary due to differing methodologies, the convergent trend across these independent sources strengthens the credibility of our central finding of substantial progress against VAD in China. Further findings indicate that, even with significant reductions in age-standardized incidence and prevalence rates over the past thirty years, VAD continues to pose a major public health challenge in 2021. This is evidenced by ongoing sex disparities and a bimodal age distribution, with peaks in children under 14 years and young adults aged 20–34 years. Critically, temporal trends revealed a pivotal inflection point around 2013, where the historically higher burden in males reversed, with females exhibiting significantly higher incidence and prevalence thereafter. Although China surpassed the global average in reducing the VAD burden, the increasing gender disparity and the concentration of the burden in vulnerable age groups necessitate targeted interventions.

The reversal of sex disparities around 2013 marks a key epidemiological shift. Prior to this period, males consistently bore a higher burden of VAD, likely linked to higher physiological demands during growth periods and socioeconomic factors privileging male nutrition in earlier decades. The post-2013 female-predominant burden may reflect complex nutritional transitions driven by rapid urbanization. Studies indicate that urban dietary shifts in China have reduced traditional vitamin A-rich food sources (e.g., animal liver, dark leafy vegetables) among young women, compounded by increased micronutrient needs during reproductive ages (27, 28). From a policy perspective, the period around 2012–2013 saw the deepening of initiatives like the National Nutrition Improvement Program for Rural Students, which provided nutritional subsidies that may have disproportionately benefited school-aged boys, potentially narrowing their gap with girls (29). Concurrently, nationwide supplementation programs initially prioritized children, potentially overlooking adolescents and young adults (30). The observed plateauing of declines after 2013 suggests saturation of past interventions, necessitating sex-stratified strategies-particularly for females aged 20–34, who exhibit the highest incidence.

Distinct age-specific vulnerabilities emerged. Children under 14 years, especially males, experienced the highest DALY burden, underscoring VAD’s severe impact on childhood morbidity (e.g., xerophthalmia, immune impairment) (31–33). This aligns with global evidence linking early-life VAD to irreversible visual deficits and heightened infection-related mortality (34). Conversely, the bimodal peak in young adults (20–34 years), with females disproportionately affected, highlights life-stage risks. Women in this cohort face increased vitamin A demands during pregnancy/lactation, while dietary inadequacies may persist due to weight-conscious diets or limited access to diverse foods (35). The convergence of high DALYs in children and high incidence in young adults signals an intergenerational cycle of malnutrition, where maternal VAD perpetuates deficiency in offspring.

Globally, VAD burden declined significantly, yet extreme inequities persist. Sub-Saharan Africa accounted for the highest incidence across all age groups, with children under 5 most severely affected-a stark contrast to China’s trajectory. Our frontier and correlation analyses robustly linked SDI to reduced VAD burden: higher SDI correlated with lower age-standardized rates across 204 countries. This emphasizes that economic growth, fortified by food fortification programs and primary healthcare access, drives VAD reduction (36–38). However, China’s faster decline compared to global averages suggests synergistic effects of targeted policies (e.g., supplementation, dietary diversification), though regional disparities within China require further exploration (39).

Bayesian projection models indicate continued declines in VAD incidence through 2040, but with concerning divergences. The burden is expected to decrease more rapidly in females, widening the sex gap and leaving males with persistently higher incidence. This trend mirrors patterns observed in other nutrition-related deficiencies and may reflect slower penetration of interventions among male populations. The model’s projection is robust in the sense that it is based on a strong, consistent historical trend. However, these forecasts are contingent on the continuation of current socioeconomic and public health trajectories. They would be sensitive to major, unanticipated policy shifts (e.g., a new, highly effective, sex-targeted supplementation program) or large-scale societal changes that alter dietary patterns differentially by sex. To avert this trajectory, interventions must address sex-specific determinants: for example, integrating vitamin A supplementation into adolescent health programs for girls and leveraging school-based platforms for boys.

This study has limitations that must be considered when interpreting the results. Limitations include those inherent to the GBD study methodology and data sources. It is important to note that the GBD estimates are synthetic, modeled data that aim to create comparable estimates across geography and time, rather than direct primary measurements. While this allows for robust temporal and cross-country comparisons, the estimates for specific subnational populations within China should be interpreted with caution, as they are influenced by the availability and quality of underlying data sources. Despite significant progress, VAD remains a public health challenge in China, characterized by persistent sex and age disparities. Projections further indicate a concerning trend where males are expected to maintain higher incidence rates than females moving forward. Also, and importantly, our analysis is conducted at the national level. As the reviewer rightly points out, China exhibits substantial socioeconomic and health disparities across its regions (e.g., coastal vs. inland, urban vs. rural). The GBD 2021 study does provide estimates at the provincial level for China. However, a detailed subnational analysis was beyond the scope of this present paper, which aimed to provide a comprehensive national overview and global context. The absence of regional stratification is a limitation of our current study, as it precludes the identification of high-burden hotspots within China that might require targeted policy action. We explicitly acknowledge this limitation and recommend that future research leverages GBD’s subnational data for China to uncover these critical internal disparities and inform more precise, region-specific nutritional interventions. Globally, the burden reveals stark and unacceptable inequities, heavily concentrated in regions like Sub-Saharan Africa. Moving forward, effective action must address the distinct epidemiological patterns revealed.

In conclusion, while China has made commendable strides in reducing VAD burden over three decades, significant challenges persist. The evolving sex disparity, the concentrated burden among young children and young adults, and the stark global inequalities demand focused attention. Sustained, targeted interventions addressing these specific vulnerabilities, coupled with intensified global cooperation in high-burden areas, are paramount. Concerted action guided by these insights is crucial to finally mitigate VAD’s impact as a major public health concern globally.

## Data Availability

The original contributions presented in the study are included in the article/supplementary material, further inquiries can be directed to the corresponding author/s.
